# SARCOIDOSIS WITH PHOTOSENSITIVE LESIONS: A RARE VARIANT

**DOI:** 10.4103/0019-5154.49004

**Published:** 2009

**Authors:** Asok Gangopadhyay, Jayanta Kumar Das, Sujata Sengupta

**Affiliations:** *From the Department of Dermatology, R.K.M Seva Pratisthan and Vivekananda Institute of Medical Sciences, Kolkata, India*; 1 *From the Department of Dermatology, B P Poddar Hospital and Research Centre, Kolkata, India. E-mail: senguptasujata@yahoo.co.in*

## Case 1:

**Figure 1:**
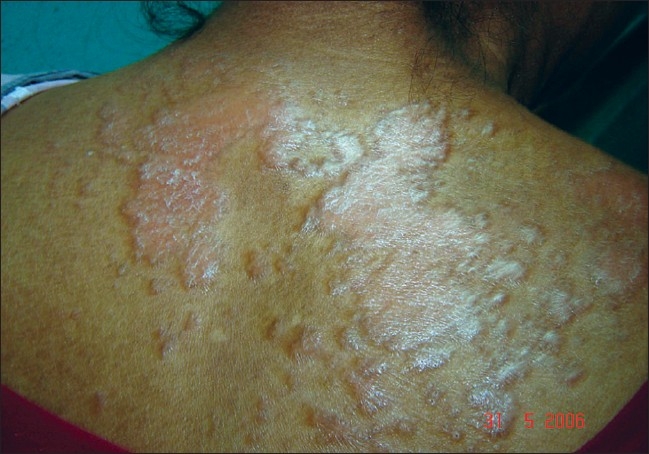
Papules and coalescent plaques on the exposed areas of the back

**Figure 2:**
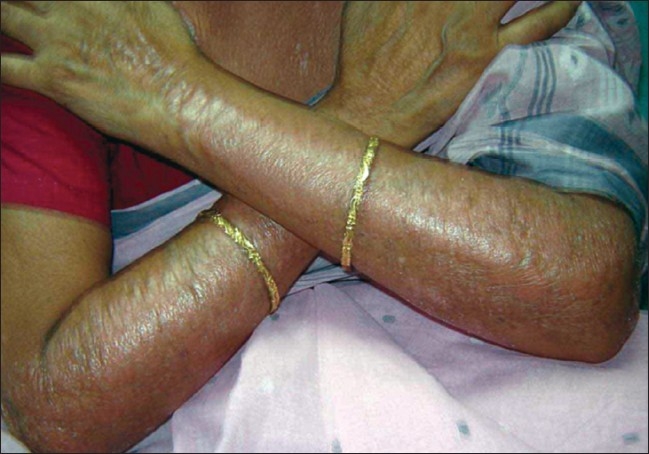
Lesions on extensor aspect of forearms

**Figure 3:**
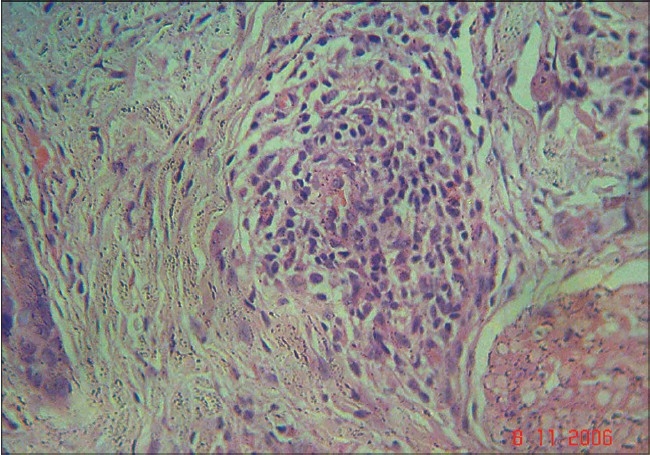
Non-caseating epithelioid granuloma with sparse lymphocytes and occasional giant cells (H and E, 40×)

**Figure 4:**
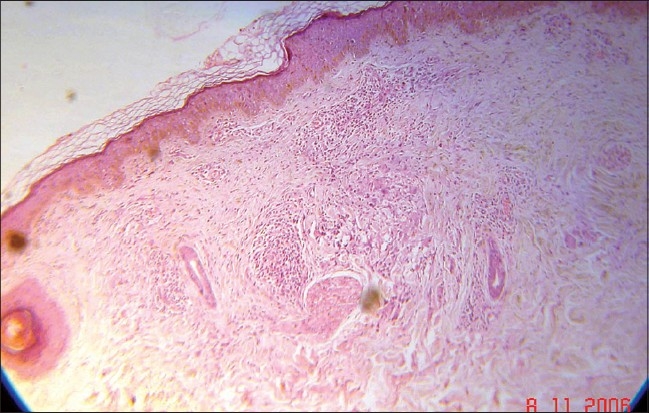
Sarcoid granuloma in Case 1 (H and E, 10×)

**Figure 5:**
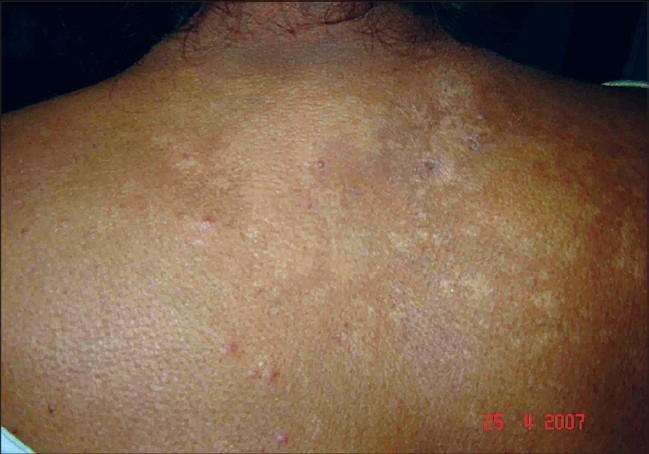
Post treatment photographs of Case 1

**Figure 6:**
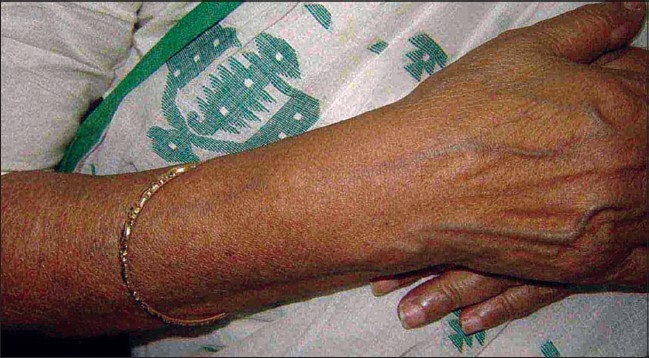
Clearing of lesions after nine months

A 63-year-old nurse presented with mildly pruritic papules and coalescent plaques on the forehead, extensor aspect of arms, and upper back [Figure 1, Figure 2]. On exposure to the sun, she experienced mild redness and a burning sensation in all her skin lesions. There was no fever, dyspnea, joint pain or oral ulcers. Systemic examination was normal. Tests revealed mild anemia (11 mg%), raised ESR (50 mm), negative Mantoux reaction and antinuclear antibody. Biopsy of a papule from the back showed epithelioid granuloma with sparse lymphocytes, occasional giant cells and no caseation [Figure 3, Figure 4]. Subsequent investigations revealed raised serum angiotensin converting enzyme (SACE, 128 U/L), serum calcium 9.8mg/dl, and normal chest radiograph, pulmonary and liver functions. Ophthalmoscopic evaluation was normal. The final diagnosis was cutaneous sarcoidosis with photosensitive lesions. She was given oral prednisolone (30mg/day, tapered off by two weeks) and hydroxychloroquine sulphate (HCQS, 200mg/day) along with sunscreens. This produced excellent results by four months with almost complete clearing of the skin lesions within nine months [Figure 5, Figure 6].

## Case 2:

**Figure 7:**
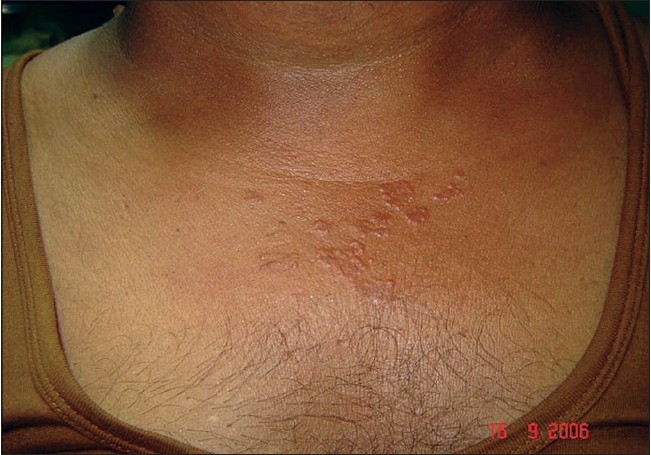
Papular skin lesions on upper chest in Case 2

**Figure 8:**
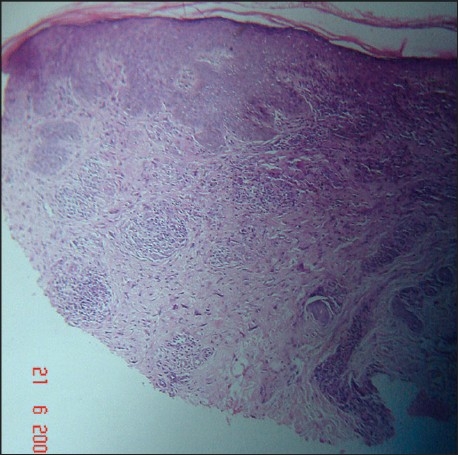
Multiple granulomas in dermis in Case 2 (H and E, 10×)

**Figure 9:**
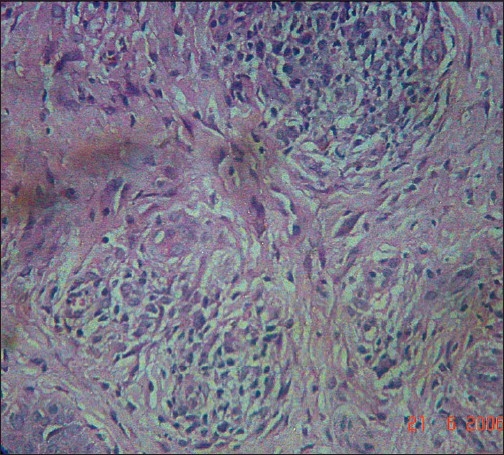
Sarcoid granuloma in Case 2 (H and E, 40×)

**Figure 10:**
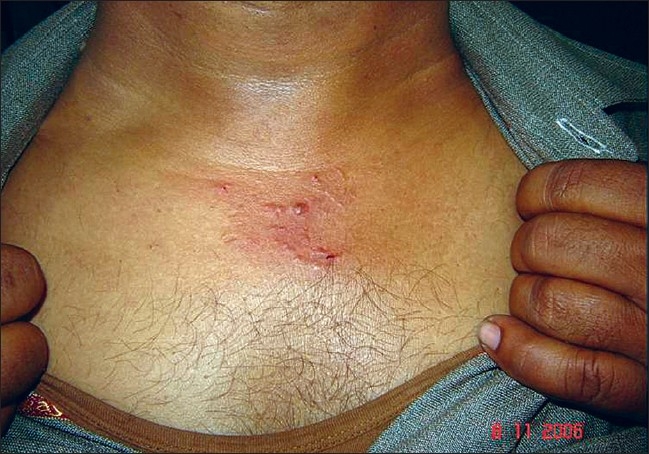
Case 2 after three months of therapy

A 37-year-old businessman suffered from asymptomatic skin colored papules in the upper chest for six months [Figure 7]. Sun exposure made them pruritic and erythematous. The rest of the dermatologic and systemic examination was normal. Investigations showed raised ESR (45 mm), negative Mantoux test and normal chest X-Ray. Typical sarcoid granuloma was seen in histopathology [Figure 8, Figure 9]. SACE was raised (90U/L) and serum calcium was normal. Other relevant tests done were also normal. Sunscreens, topical steroids and hydroxychloroquine sulphate (200mg EOD) produced significant improvement within three months [Figure 10].

Polymorphic disease pattern and lack of definite diagnostic tests make sarcoidosis a disease of exclusion. Late lesions of polymorphic light eruption (PMLE) are usually scaly or lichenified, unlike the firm fleshy look in Case 1. Moreover, histology and biochemistry ruled out PMLE. We had also considered lupus erythematosus in this case, but other clinical and serological features were absent. Sarcoid lesions mimicking lupus have been reported before.[5] Case 2 closely resembled granuloma annulare, but photosensitivity, negative Mantoux test, classical naked granuloma without any necrobiosis and raised SACE helped to exclude it.

In a recently published study of 23 Indian sarcoid patients, photosensitive skin lesions have not been documented.[6] The preferential distribution of skin lesions in photo-exposed areas and the significant clearing with hydroxychloroquine sulphate are the two aspects that make the cases special. A French article describes a Tunisian woman with papular erythema of the face, unresponsive to topical steroids.[7] She was histologically diagnosed to be a case of cutaneous sarcoid and the authors opined that photo-induced sarcoid is a distinct entity. Our cases further strengthen the view.
